# The Effect of Computer Usage in Internet Café on Cigarette Smoking and Alcohol Use among Chinese Adolescents and Youth: A Longitudinal Study

**DOI:** 10.3390/ijerph9020496

**Published:** 2012-02-06

**Authors:** Liyun Wu, Jorge Delva

**Affiliations:** School of Social Work, University of Michigan, 1080 S. University Ave., Ann Arbor, MI 48109, USA; Email: jdelva@umich.edu

**Keywords:** computer, internet café, smoking, drinking, adolescents and youth, China

## Abstract

We used longitudinal data to investigate the relationship between computer use in internet cafés and smoking/drinking behavior among Chinese adolescents and young adults. Data are from two waves of the China Health and Nutrition Survey (2004 and 2006). Fixed effects models were used to examine if changes in internet café use were associated with changes in cigarette smoking and drinking of alcohol. Male café users spent on average 17.3 hours in front of the computer/week. This was associated with an increase in the probability of being a current smoker by 13.3% and with smoking 1.7 more cigarettes. Female café users spent on average 11 hours on the computer/week. This was associated with an increase in the probability of drinking wine and/or liquor by 14.74% and was not associated with smoking. Internet cafés are an important venue by which adolescent and young adults in China are exposed to smoking and drinking. Multi-component interventions are needed ranging from policies regulating cigarette and alcohol availability in these venues to anti-tobacco campaigns aimed at the general population but also at individuals who frequent these establishments.

## 1. Introduction

It has been widely recognized that the diffusion of technology, as represented by the widespread use of computers in the workplace, plays a crucial role in national economic growth and development [1,2]. However, the impact of computer use on the development of youth and adolescents health is mixed. Using data from the 2001 Current Population Survey (CPS) computer use supplement, Fairlie [3] found an association between home computer access and higher likelihood of enrollment in school. Using the 1988 National Educational Longitudinal Survey (NELS-88), Attewell and Battle [4] showed a positive impact of home computer use on students’ math and reading scores. In contrast, Fuchs and Woessmann [5] found that home computers had a negative effect on math and reading scores after controlling for observable characteristics of students, families, and schools. Malamud and Pop-Eleches [6] conducted a field experiment in Romania where vouchers for the purchase of home computers were provided to low-income children. Their findings were mixed: students that won vouchers received lower school grades in math, English, and Romanian, but higher scores in tests of computer use. Finally, Angrist and Lavy [7] found no effect from computer use on math test scores in Israeli schools. However, none of the above studies examined the potential effects of computer use on health. 

Computer use in China is quite different from that in industrialized countries. Since relatively few Chinese owned computers at home in the early 2000s, most youths went to internet cafés to get online and play games [8,9]. As a result of what appeared to be an alarming rise in the number of youths spending time in internet cafés, governments at all levels initiated regulations to curb illegal internet cafés and censor unhealthy online information [10]. However, driven by profits, many internet cafés still operated illegally and attracted young customers with very low costs and availability of longer opening hours [11]. A key concern regarding the health impact of internet cafés usage is the extent to which their use may be associated with cigarette smoking and alcohol consumption among Chinese adolescents and youth. 

Conceptually, substantial computer use in cafés may encourage the formation of social networks that promote negative behavior. Studies have found that adolescents attempt to disassociate from their parents and spend more time with their peers in their teenage years [12]. The formation of adolescent peer groups could be caused by many different factors: attending the same class, living in the same neighborhood, being relatives or family connections, or having the same opinions or behaviors. As a result of spending time together, individuals tend to imitate their behaviors based upon their peers–playing the same sports and games, eating the same types of food, visiting the same websites, and sharing many other habits. The shared behaviors between an individual and peers may directly or indirectly affect his/her health status. Unsupervised adolescents in internet cafés may imitate the behavior of their peers by smoking heavily, drinking alcoholic beverages, continuously playing computer games, and surfing the same internet web pages. Most of these shared behaviors appear to be detrimental to health, and are more likely to occur in the café setting. In sharp contrast, those adolescents not visiting internet cafés may have healthier behaviors since parents can better supervise and monitor youth behaviors and support more positive ones. 

It is well documented that widespread smoking has been a serious public health problem in China for a number of years [13]. This is particularly the case for adolescents who because of their earlier exposure to smoking may experience worse health consequences overtime. Even if individuals in internet café do not smoke, they are exposed to a considerable amount of secondhand smoking. 

Besides cigarette smoking, alcohol use has also increasingly become a serious public health problem in China. Drinking has been shown to occur among 69 percent of Chinese adolescents and 66.1 percent among those 18 years and older [14,15]. These are concerning rates as alcohol consumption is one of the contributing factors to chronic diseases, accidental injuries, and intimate partner violence [15,16,17]. With the growing number of internet cafés, it appears that alcohol has become a common beverage among the younger generation who frequent these settings [18]. In light of these public health concerns, the purpose of this study was to prospectively examine if changes in internet café usage were associated with changes in cigarette and alcohol use among Chinese adolescents and young adults. 

## 2. Data and Methods

### 2.1. Sample

Data are from the 2004 and 2006 waves of the China Health and Nutrition Survey (CHNS), administered by the Population Center at UNC Chapel Hill. The CHNS is a longitudinal survey with a sample of approximately 4,400 households with 16,000 individuals including newborns to older individuals drawn to represent nine of thirty-one provinces in China, including two rich coastal provinces, five middle-income provinces and two poor interior provinces. A multistage and random cluster sampling procedure was used to select cities, counties, neighborhoods, villages and households. 

For this study, we restricted the sample to adolescents and young adults ages 15–30 years old in 2004 or 2006. We restricted the sample to this age group for conceptual and empirical reasons. Conceptually, at age 15, Chinese adolescents begin high school and thus represent an important transition from middle school to a period when they acquire considerable independence. Empirically, our analyses of the CHNS indicated that very few youth under age 15 had ever engaged in the behaviors of interest (e.g., only 2.6% of 14 year olds and younger had visited an internet café, only two had ever smoked). As far as the upper age constraint, conceptually we restricted the sample to 30 year olds because youth are expected to become independent at the age of thirty according to Chinese culture. Empirically, the percent of individuals 31 years and older that had visited an internet café was very small (only 1%) compared to those 25–30 years of age (around 5%). Our study consisted of 1,007 male respondents and 927 female respondents. Due to sample attrition, we eventually include 2,484 observations in the final analysis.

### 2.2. Measures

The CHNS includes questions on computer and internet café usage as well as on cigarette and alcohol use. We also used data from the restricted community-level dataset. Variables are described next. 

*Cigarette Smoking*. Smoking was assessed by first asking “Have you ever smoked cigarettes (including hand-rolled or device-rolled)?” Those who answered ‘Yes’ were then asked “Do you still smoke cigarettes now?” Using these data we generated one dichotomous indicator of smoking-current smoker (1 = Yes, 0 = No, which includes never smokers and non-current smokers. We also created one continuous variable representing the total number of cigarettes smoked per day as the CHNS asked current smokers for the number of cigarettes they smoke per day. 

*Alcohol Consumption*. For each of three types of alcohol beverages–beer, grape wine (including various colored wines, rice wine), and liquor-survey participants were asked “Do you drink this type of alcohol?” If they answered ‘Yes’ they were then asked “How much do you drink each week?” and had to indicate the number of beer bottles and the total amount of grape wine and/or liquor according to the Chinese unit of measurement “liang” (1 liang, 2 liangs, 3 liangs, *etc*.) (Note: 1 liang is equivalent to 50 grams or 1.7637 ounces.)

Because in China beer consumption is most common among the younger generation while wine (including various colored wines and rice wine) and liquor are more often consumed by older adults, we created two separate dependent variables: a dummy-coded variable for current drinking of beer and another for current drinking of wine and/or liquor. 

*Computer Use and Internet Café Usage*. Although the questions asked about computer use and internet access in the 2004 and 2006 surveys were slightly different, we created categories of use that would be equivalent in both survey years. Essentially, study participants were asked to indicate if they presently use a computer to (a) ‘surf the internet’; (b) ‘participate in chat rooms’; and/or (c) ‘play computer games, *etc*.’ and for those who answered affirmatively, the number of hours they spend using a computer for each of the three types of uses on a typical day during the week (Monday–Friday) and on weekends (Saturday and Sunday). These data were used to construct a continuous variable with zero indicating no computer use and a positive number indicating the total number of hours for the entire week (5 × weekday + 2 × weekend). In addition, using the questions that asked participants to indicate if they have access to the internet and where they use the computer to access the internet, we created three dummy-coded variables to distinguish three different types of computer users: (1) Users in internet café only-individuals who only have access to internet in a café and do not own a computer at home; (2) Users at home only-individuals who access the internet at a home-based setting such as at their home, a friend’s or relative’s home, or in school; and (3) Users of both internet cafés and home-based. 

*TV-Based Screen Hours*. A continuous variable was created to measure the total TV-based screen hours consisting of aggregated hours individuals spent in three sedentary activities in a typical week-watching TV, DVDs, playing video games. These variables were included as controls in the analyses. By statistically controlling for screen hours we distinguished the effects of computers and televisions, and examined if computer use substituted or complemented the TV-based screen hours.

*Demographic Characteristics*. We included as controls the following continuous variables–age and age squared–and the following categorical variables–minority status (1 = yes—corresponds to anyone who self-identifies as non-Han such as Mongolian, Tibetan, *etc.*, 0 = no), education (1 = elementary or below, 2 = junior high school, 3 = high school or equivalent, 4 = college or above), marital status (1 = married, 0 = not married), whether the individual presently is in school (1 = yes, 0 = no), and whether the individual is working (1 = yes, 0 = no). 

*Household-Level Characteristics*. Three variables were included. They are: (1) a dummy-coded variable for living in urban site; (2) a continuous variable measuring household size; and (3) the log value of household gross income *per capita*. 

*Community-Level Characteristics*. We also included a dummy-coded variable to measure whether the local community has access to cable TV. We included this variable to control for the potential effect of cigarettes and alcohol advertisements shown on TV. We also included a dummy-coded variable for the province where the participants resides at the time of the interview to control for the time-invariant characteristics at the provincial level. 

### 2.3. Analysis

The longitudinal nature of the study enabled us to use fixed effects (FE) models to examine if changes in internet café use would be associated with changes in cigarette and alcohol consumption over a period of two years. Because the percentage of males and females who are smokers and drinkers are so different, we thought it important to conduct the analyses separately by gender. In detail, the percentages of currently smoking among males and females are 38% and 1%, respectively; the percentages of presently drinking beer among males and females are 37% and 5%, respectively; the percentages of presently drinking wine and/or liquor among males and females are 26% and 4%, respectively. 

The FE model serves to difference out any time invariant unobservable factors, something that is omitted when using ordinary least squares (OLS) regressions. Therefore, the FE model produces more reliable point estimates and is an improvement over standard OLS models. 

We begin with a simple relationship between internet café use and cigarette/alcohol consumption:


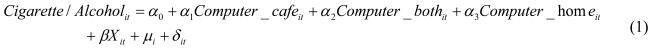


The dependent *cigarette/alcohol variables* are dichotomous, with a value of 1 if the youth *i* at time *t* consumed the particular substance and a value of zero if the participant did not consume. As there are four cigarette/alcohol dependent variables, namely, currently smoking, total # cigarettes smoked per day, currently drinking beer, and currently drinking wine and/or liquor, we ran four separate regressions with these four different dependent variables. Since the smoking and drinking participation differs considerably by gender, the regressions were run separately for males and females. 

The key independent variables of interest were computer use hours by location. We were particularly interested in exploring the effect of computer use in internet cafés. Thus, we distinguished computer use into three types-in café only, at home only, and in both settings. In Equation (1) *computer_café_it_* is computer use hours only in cafés by person *i* at time *t*, *computer_home_it_* is computer use hours only at home-based setting, and *computer_both_it_* is computer use hours in both settings. *α_1_* is the parameter of our interest, estimating the health effect of computer use in café. *X_it_* includes a set of covariates, including individual’s age, age square, gender, minority status, educational status, whether in school, whether working, urban residency, household size, logarithm of per capita household gross income, community-level cable TV indicator, and provincial dummies. 

The error terms in Equation (1) is composed of two components: *μ_i_* corresponds to the individual-level fixed effect which accounts for the time-invariant unobserved individual characteristics and *δ_it_* captures the idiosyncratic errors. 

As is well-known, the OLS estimate of *α_1_* will be biased if the individual fixed effect *μ_i_* is correlated with *computer_cafe_it_*. Since access to internet café is not random and is related to unobservable characteristics such as individuals’ personality, such bias is very likely. With the longitudinal sample we were able to remove the bias resulting from the omitted factor *μ_i_ by* differencing the Equation (1) across the two waves. The regression below shows the difference equation which is estimated in the FE approach: 


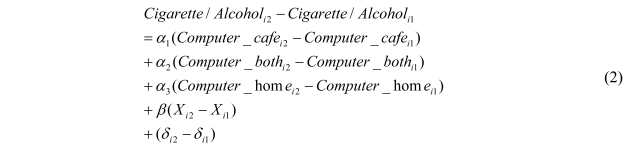


To allow for causal interpretation of the parameter 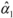 and make it a consistent estimate of café use on smoking/drinking, we assumed Cov((*δ*_i2_ − *δ*_i1_), (*computer_cafe*_i2_ − *computer_cafe*_i1_)) = 0. Heteroskedasticity-robust standard errors are reported. 

## 3. Results and Discussion

### 3.1. Computer Use Patterns

**Figure 1:**
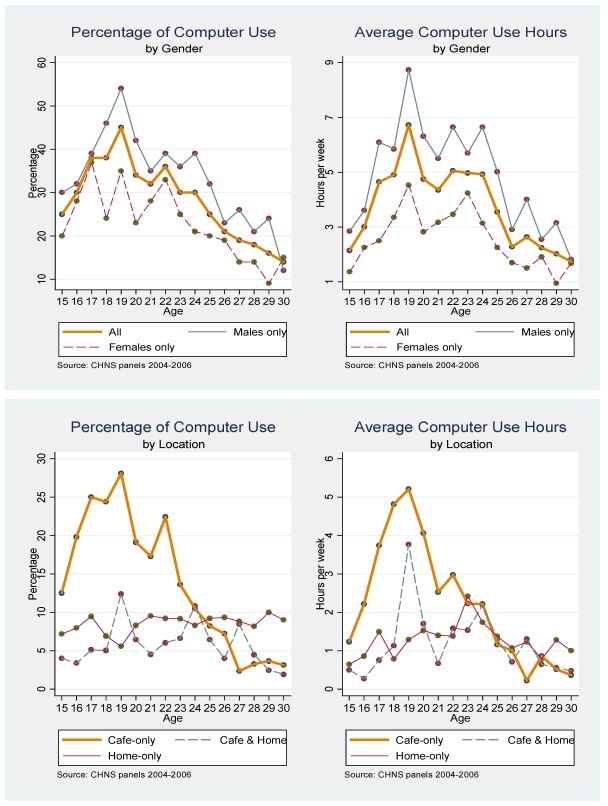
Distribution of total computer use and internet café usages by age and gender among 15–30 year old Chinese (percentage and average hours per week): 2004 and 2006 pooled CHNS data.

The top left-hand panel in Figure 1 indicates that the highest percent of computer use (in any location) occurs among 19 year olds (45%) with more than 30% of 17–24 year olds reporting using computers during the week, *versus* less than 30 percent of individuals in other ages. The participation rate declines monotonically for individuals 25 years old and older. Computer use is considerably greater among males than females, regardless of age. As shown in the top right-hand panel, 19 year olds spend the most time using computers (~7 hours/week) and 17–24 year olds spend an average of 5 hours in front of the computer screen every week. Males on average spend one more hour on the computer than females irrespective of age. 

The two panels on the bottom show the participation rate and average use hours per week for three types of users. The lower left-hand panel displays that the participate rate of computer use among café-only users 24 or younger is around 20 percent while the rate is fluctuating between 5 and 10 percent among both users and home-only users. The lower right-hand panel presents the average hours/week among three types of users. Among individuals aged 24 or younger, they spend more hours in café-only setting than other two settings. The trend is indistinguishable for all users older than 24. 

As shown in Table 1, a greater percent of computer users than non-computer users had ever smoked (25.60% *vs.* 21.76%) and were current smokers (24.24% *vs.* 20.04%) but no differences were observed in the average number of cigarettes smoked/day. A considerably larger percent of computer users than non-computer users consumed beer (33.19% *vs.* 19.63%) and wine/liquor (20.68% *vs.* 15.12%). 

For the computer user subsample, the average time spent using the computer was as high as 14.80 hours/week (equivalently, about 2 hours/day). Apparently, computer users spend less time watching television and more time playing DVDs and video games. When compared to non-computer users, users are younger, males, non-minority, have a high school education or higher, live in urban areas, have higher incomes, and live in households with smaller number of people. 

**Table 1:** Descriptive statistics for pooled sample of males and females aged 15–30 years old: 2004 and 2006 CHNS.

		Using Computer (N = 703)		Not Using Computer (N=1781)	
Variable List	Mean	Std Dev	Mean	Std Dev	T-test ( *p*-value)
Smoking					
% ever smoked	25.60	0.44	21.76	0.41	0.04
% currently smoking	24.24	0.43	20.04	0.40	0.02
#cigarettes smoked	2.88	6.40	2.93	6.71	0.87
Drinking					
% drink beer	33.19	0.47	19.63	0.40	0.00
% drink wine and/or liquor	20.68	0.41	15.12	0.36	0.00
Screen-based Media Use Hours					
Avg computer use hours/week	14.71	11.80	--	--	--
Avg TV hours/week	13.91	9.21	14.78	8.80	0.03
Avg DVD + Video hours/week	8.97	6.77	8.23	6.23	0.15
Avg TV + DVD + Video hours/week	17.04	10.68	16.30	9.64	0.10
Demographics					
Age	22.33	4.65	24.41	5.01	0.00
Age squared	520.59	213.02	621.07	235.19	0.00
Female = 1	0.39	0.49	0.52	0.50	0.00
Minority = 1	0.06	0.24	0.15	0.36	0.00
Youth Education---					
Elementary or below = 1	0.01	0.10	0.16	0.37	0.00
Junior high school = 1	0.26	0.44	0.59	0.49	0.00
High school or equivalent = 1	0.45	0.50	0.23	0.42	0.00
Bachelor or above = 1	0.27	0.45	0.02	0.14	0.00
Urban = 1	0.44	0.50	0.16	0.37	0.00
Household size	3.89	1.29	4.49	1.44	0.00
HH income gross (CPI-adjusted)	37,204.52	32,454.59	25,399.97	26,281.06	0.00
HH income per person (CPI-adjusted)	9,450.56	8,535.89	5,012.88	5,064.60	0.00
Community has cable TV = 1	0.87	0.34	0.60	0.49	0.00

### 3.2. Impact of Computer Use and Internet Café Usage on Cigarette Smoking

Table 2 presents the results of the fixed effects analyses to estimate the association of computer usage with two smoking indicators: the likelihood of currently smoking, and the total number of cigarettes smoked/day. Since the prevalence rate of smoking among females was only 1 percent, results are presented separately by gender. Because none of the estimated associations were statistically significant for females, the results discussed below correspond to the male findings. 

**Table 2:** Relationship of computer use (in Internet Café *vs.* NOT in Internet Café) with the probability of being a current smoker, and the total number of cigarettes smoked per day among adolescents and young adults in China: 2004 and 2006 CNHS.

Variables	Currently Smoking (0/1)		Total # cigarettes smoked/day	
	Males	Females	Males	Females
	(1)	(2)	(3)	(4)
**Computer hours/week in café only**	0.0077 **	0.0024	0.0978 **	0.0113
	(0.0025)	(0.0024)	(0.0336)	(0.0123)
**Computer hours/week both in café and at home**	0.0050	0.0002	−0.0100	0.0005
	(0.0038)	(0.0004)	(0.0529)	(0.0020)
**Computer hours/week at home only**	0.0058	−0.0002	0.0854	−0.0015
	(0.0032)	(0.0004)	(0.0528)	(0.0022)
Total screen hours (TV+DVD+VIDEO)	−0.0015	0.0002	0.0078	0.0002
	(0.0013)	(0.0002)	(0.0208)	(0.0010)
Age	0.0316	−0.0078	0.4041	−0.0768
	(0.0778)	(0.0177)	(1.2538)	(0.0932)
Age squared	−0.0005	0.0000	−0.0033	0.0010
	(0.0015)	(0.0003)	(0.0256)	(0.0015)
Marital status	0.1639	0.0090	2.9413	0.0244
	(0.1152)	(0.0119)	(1.9528)	(0.0586)
Whether in school?	−0.1179	−0.0104	−1.5866	−0.0692
	(0.0958)	(0.0086)	(1.7436)	(0.0466)
Whether working?	0.1580 **	−0.0261	3.0323 **	−0.1083
	(0.0554)	(0.0187)	(1.1240)	(0.0822)
Education: middle school	−0.1838	0.0144	−3.2583	0.0571
	(0.2454)	(0.0110)	(7.6075)	(0.0511)
Education: high school or equivalent	−0.2959	0.0225	−6.0069	0.0881
	(0.2752)	(0.0153)	(8.1722)	(0.0804)
Education: bachelor or above	−0.2521	0.0195	−6.2184	0.0748
	(0.3298)	(0.0248)	(8.8027)	(0.1298)
Household size	−0.0442	−0.0012	−0.5170	0.0025
	(0.0337)	(0.0078)	(0.6923)	(0.0388)
Log(income)	−0.0417 *	0.0002	−0.3383	0.0031
	(0.0183)	(0.0018)	(0.3018)	(0.0094)
Community-cable TV	0.0199	0.0253	−0.1548	0.1857
	(0.0390)	(0.0173)	(0.6549)	(0.1067)
Constant	0.5643	0.1547	4.0813	1.1221
	(0.9869)	(0.2317)	(15.3959)	(1.2276)
Observations	1,289	1,195	1,273	1,195
R-squared	0.1102	0.0568	0.0876	0.0560
# of persons	1,007	927	1,001	927

Notes: Rows with numbers in parenthesis (1), (2), and so forth in the headings of the table are utilized to indicate columns to facilitate the description of results in the body of the manuscript. Numbers inside the table that are in parentheses indicate robust standard errors. * *p* < 0.05; ** *p* < 0.01.

Columns 1–2 present the results of the effects of computer use on the probability of current smoking. For males (Column 1), the magnitude of the FE estimate indicates that one hour of time spent in café only can increase the probability of currently smoking by 0.77%. Since male café-only users on average spend 17.3 hours in cafés each week, the café-only computer access can lead to an increase in the probability of smoking by 13.3% on a weekly basis, a substantial amount. The estimates for home-only and both settings also were not statistically significant. 

Columns 3–4 present the results of the effects of computer use on the total number of cigarettes smoked per day. For males (Column 3) each hour of computer use in café only can increase the total number of cigarettes smoked by 0.09 units, equivalent to 1.7 units (or almost two cigarettes) on a weekly basis. 

As for the covariates, working status was a strong predictor for males’ currently smoking, the risk increasing by 15.8% if the respondent was currently employed. Per capita household income was significantly and inversely associated with current smoking. None of the estimates were significant for females. 

### 3.3. Impact of Computer Use and Internet Café Usage on Alcohol Consumption

Table 3 presents the FE estimates of computer usage on the likelihood of drinking beer, and wine/liquor by gender. Changes in computer usage in internet cafes or elsewhere were not significantly associated with beer consumption, among males and females. Among men, age and educational attainment were strong predictors determining the probability of drinking beer among them. The age effect turned out to be non-linear and quadratic indicating that beer consumption increases at a slower rate with age. Educational attainment was inversely associated with beer consumption. However, females displayed a different pattern. Total TV-based screen hours were associated with an increase in the incidence of drinking beer. One hour of TV-based screen time can increase the beer consumption by 0.24% among females, an amount equivalent to about 4.08% higher rate of drinking beer on a weekly basis with 17 hours spent watching television. Household size also appears to play an important role in determining the probability of drinking beer among females. Females drink less beer if they live with more family members. 

The two models on the right columns (Table 3) provide the results of the effects of computer use on drinking wine/liquor by gender. Although there is lack of association between computer use and drinking wine/liquor among males, there is a statistically significant finding for females, showing that one hour in café only can predict 1.34% higher incidence of drinking wine/liquor. On a weekly base, females on average spend 11 hours in internet café, which is equal to a 14.74% higher rate of drinking wine/liquor. Although the estimate for computer use in both settings was also statistically significant at the 0.05 level, its magnitude is smaller and the estimate for home use is no longer significant. These three estimates altogether reinforce the finding that café plays a risk factor determining the probability of drinking wine/liquor among females. Larger household size also appears to be a protective factor, reducing the rate of drinking wine/liquor among females.

**Table 3:** Relationship of computer use (in Internet Café *vs.* NOT in Internet Café) with the probability of currently drinking beer, wine and/or liquor among adolescents and young adults in China: 2004 and 2006 CHNS.

	Beer (0/1)		Wine and/or Liquor (0/1)	
Variables	Males	Females	Males	Females
	(1)	(2)	(3)	(4)
**Computer hours/week in café only**	−0.0031	0.0102	0.0006	0.0134 *
	(0.0044)	(0.0069)	(0.0038)	(0.0068)
**Computer hours/week both in café and at home**	0.0018	0.0093	−0.0026	0.0100 *
	(0.0047)	(0.0050)	(0.0049)	(0.0049)
**Computer hours/week at home only**	0.0085	−0.0006	0.0046	0.0058
	(0.0051)	(0.0040)	(0.0033)	(0.0031)
Total screen hours (TV+DVD+VIDEO)	0.0021	0.0024 *	0.0019	0.0013
	(0.0016)	(0.0012)	(0.0013)	(0.0008)
Age	0.2301 *	0.0454	0.0331	−0.0115
	(0.0988)	(0.0619)	(0.0873)	(0.0511)
Age squared	−0.0040 *	−0.0009	−0.0003	0.0002
	(0.0019)	(0.0012)	(0.0017)	(0.0009)
Marital status	0.1599	0.1077	0.0617	0.0899
	(0.0966)	(0.0893)	(0.1275)	(0.0893)
Whether in school?	0.0286	0.0718	−0.0698	0.0110
	(0.1009)	(0.0810)	(0.0905)	(0.0662)
Whether working?	−0.0021	0.0221	0.0633	−0.0079
	(0.0658)	(0.0362)	(0.0671)	(0.0196)
Education: middle school	−0.4061 *	0.0189	0.1294	0.0112
	(0.1691)	(0.0293)	(0.1235)	(0.0300)
Education: high school or equivalent	−0.4709 *	0.0350	−0.1618	0.0276
	(0.2126)	(0.1050)	(0.1765)	(0.0798)
Education: bachelor or above	−0.4419	−0.3208	−0.1028	−0.1089
	(0.2445)	(0.2745)	(0.2211)	(0.1135)
Household size	−0.0181	−0.0484 *	0.0159	−0.0747 **
	(0.0298)	(0.0222)	(0.0341)	(0.0265)
Log(income)	0.0129	−0.0019	0.0054	−0.0022
	(0.0202)	(0.0070)	(0.0239)	(0.0070)
Community-cable TV	−0.0776	0.0301	−0.0433	0.0447
	(0.0600)	(0.0316)	(0.0585)	(0.0240)
Constant	−2.3811	−0.3893	−0.4696	0.4097
	(1.2316)	(0.7978)	(1.1252)	(0.7148)
Observations	1,286	1,194	1,285	1,194
R-squared	0.0776	0.0848	0.0464	0.1288
# of persons	1,007	927	1,006	927

Notes: Rows with numbers in parenthesis (1), (2), and so forth in the headings of the table are utilized to indicate columns to facilitate the description of results in the body of the manuscript. Numbers inside the table that are in parentheses indicate robust standard errors. * *p* < 0.05; ** *p* < 0.01.

## 4. Discussion

This study examined if over a two-year period changes in internet café usage were associated with changes in smoking and drinking behaviors among Chinese adolescents and young adults using longitudinal data from the China Health and Nutrition Survey. We found the home computer ownership rate to be only 15% in the pooled sample with 30% of urban households having computers *versus* only 10% of rural households. Due to the relatively low rates of access to home computers, the internet café has become the primary setting for computer access. After controlling for individual, household, as well as community characteristics, results of the fixed effects regressions suggest that every hour spent in internet café can increase the likelihood of smoking by roughly 0.77% among males, and the probability of drinking wine/liquor by 1.34% among females. On a weekly basis, these two effects magnify to become 13.3% and 14.7%, respectively. 

In this study we have identified the use of computers in internet cafés as an important environmental factor that influences youth smoking and drinking behaviors. Although youth can access the computer in three types of environments-café only, home only, or both, the use of computer at home only or on both settings had no effect on smoking and drinking while the use of computers in cafés only was a strong predictor of smoking among males and drinking among females. The differences we observed in smoking/drinking among these three settings raise interesting questions regarding the extent to which characteristics of the setting itself (*i.e.*, availability of cigarettes and alcohol) and of the people who frequent these establishments (*i.e.*, more prone to engage in high-risk taking behaviors) influence the behaviors of the other patrons. More research is needed to examine the mechanisms under which the setting and the people, perhaps jointly, impact the behaviors of others.

Our findings also provide evidence of social inequality in that smoking and drinking behaviors were more common among individuals with lower education and incomes. This finding is of concern given the overall disease burden found among individuals of lower socioeconomic status. Anti-smoking and drinking interventions are needed that target the general, and particularly the more disadvantaged, populations. Another interesting finding is the role that living in larger households may play in potentially preventing females from drinking. It is plausible that these findings reflect social norms in China that place greater emphasis on preventing females from consuming alcohol and where a larger family size may serve to provide women with more supervision and monitoring further preventing alcohol consumption. 

The study findings should be considered within the context of the following three limitations. First, the CHNS does not include sampling weights due to Chinese government restrictions, preventing us from generalizing the findings to the larger population. Despite this limitation, the CHNS is a large national survey covering nine provinces with different levels of economic and developmental indicators. This wide geographic coverage enables the CHNS users to capture the enormous heterogeneity that exists in China in terms of social, economic, and behavioral characteristics. Second, since the FE estimation predicts the association between changes in the key independent variable and changes in health outcomes (smoking and drinking) net of observed and unobserved invariable characteristics of the computer user, thereby attending to the problem of endogeneity and consequently serving as an improvement over the classic OLS model. However, it should be borne in mind that FE estimates do not resolve all of the causality problems associated with observable data. For instance, if youth who smoke and drink visit internet café more often due to having a higher likelihood to engaging in risk-taking behaviors, our analysis will suffer from the bias of reverse causality, namely that changes in smoking and drinking may actually cause changes in computer access. Experimental studies are needed to address these concerns. Third, it was not possible to measure grams of alcohol consumption because the size of beer bottles in China vary considerably and the survey does not ask for the size of the bottles individuals consumed. Notwithstanding these limitations, this study, using data from a national representative longitudinal study of Chinese youth and young adults, makes a contribution to the literature by documenting how computer access in internet café affects individuals’ health. Extensive internet use is a phenomenon occurring not only in China, but also in many developing countries such as Brazil, Mexico, India, and South Africa, among others. Although there are country-specific policies in place regulating internet cafés, the policy implications based on a study of Chinese youth can also serve to shed light into other countries. 

## 5. Conclusions

Cigarette smoking and alcohol consumption are two serious, and growing, public health problems in China. Multi-component interventions (e.g., regulation of cafés, policies banning smoking) involving multiple sectors (e.g., national and local governments, internet café operators) and stakeholders (e.g., users themselves) are needed to reduce the consumption of these substances and consequently the burden of disease associated with their use. 

The study findings offer some important implications for health promotion associated with internet café usage. First, effective policies should be in place to help internet cafés become health-promoting environments. No doubt, heavy fines are not the sole solution to eliminate cafés that continue to encourage detrimental behaviors among youth. The more constructive strategy is to enact implementable policies regulating internet café operation on a daily base such as nationwide campaigns restricting smoking inside an internet café. Second, despite adolescents’ pursuit of independence, a complete lack of parental supervision could be harmful for their health suggesting that an increase in parental involvement in after-school activities, for instance, may serve to buffer against adolescents’ risky behaviors. That is, greater parental involvement in after-school activities may serve to lower usage of computers in internet cafés with the consequence of reducing youth exposure to environments where cigarettes and alcohol are easily available and their use potentially encouraged. Good educational materials on the perils of uncontrolled internet café usage should be widely distributed to local communities. Since many parents in rural China travel away from home and work as migrant workers in cities, adequate adult supervision and policies regulating internet cafés to encourage healthier behaviors are particularly pressing issues for the children who remain at home. 
